# Cultural Narratives of Micronesian Islander Parent Leaders: Maternal and Children’s Health, the School System, and the Role of Culture

**DOI:** 10.31372/20190404.1078

**Published:** 2020

**Authors:** Connie Kim Yen Nguyen-Truong, Jacqueline Leung, Kapiolani Micky

**Affiliations:** 1 Dr. Nguyen-Truong and Dr. Leung are co-first authors.; a Washington State University College of Nursing-Vancouver, Washington, United States; b Micronesian Islander Community; c Oregon State University, College of Public Heath and Human Sciences in Global Health, Oregon, United States

**Keywords:** Pacific Islanders, Micronesian Islanders, parent leaders, community-based, qualitative, capacity building, community organizing, Chuukese, Marshallese

## Abstract

*Background:* In Oregon in the United States’ Pacific Northwest, Native Hawaiians/Pacific Islanders including Micronesian Islanders (MI) substantially grew by 68%; however, research is sparse. This is often due to data aggregation as Asian and Pacific Islanders and community members’ reluctance and wariness to participate in research due to a history of unethical research in the Pacific. The MI community experienced miscarriages, stillbirths, and intellectual and developmental disabilities. Organizational MI community leaders expressed a need to explore the voices of MI parent leaders (MIPLs). The purpose of the community-based participatory qualitative descriptive pilot study was to explore the perceptions and experiences of MIPLs with maternal and children’s health, the school system, and the influence of culture. *Methods:* A trained MI community health worker recruited eight MIPLs from an urban area of the Pacific northwest in the United States. A group level assessment included illustrative storytelling and is a participant-driven qualitative method that guided data collection and analysis with real-time involvement with MIPL. The discussions lasted for 90 minutes. MIPL shared stories by writing and drawing pictures onto the flip chart papers, transcribed main points, and analyzed the data with researchers. Researchers recorded field notes of the interactions. Researchers debriefed with MIPL to assure trustworthiness and credibility of the findings. *Findings:* MIPL are Compact of Free Association citizens. Their age ranged from 26 to 42 years, have lived in the United States an average of 12.63 years, and most reported having less than $15,000 total household income before taxes. Four main themes were identified: MI cultural identity, English language and MI culture disharmony, zero or delayed prenatal care, and uncertainty for the future of MI children who have disabilities or developmentally delayed as they progress through the school system. *Conclusion:* Health care providers including nurses and school officials need to have a culturally specific understanding of the MI community and must consider their needs, culture, and language barriers.

## Introduction

The 2010 United States (U.S.) Census reveals Pacific Islanders are the fifth fastest growing racial group in Oregon in the Pacific Northwest at a 68% increase (Oregon Department of Education, 2014). The Pacific Islander community is diverse and includes: Native Hawaiian, Samoan, Tongan, Polynesian (Tahitian or Tokelauan), Guamanian or Chamorro, Micronesian Islanders, Fijian, and Pacific Islanders who have not specified a group (Multnomah County Health Department, 2015). Micronesian Islanders (MI), a minority subgroup within the Pacific Islanders, consist of people from the Federated States of Micronesia (Chuuk, Kosrae, Pohnpei, and Yap), the Republic of Marshall Islands, the Republic of Palau, Guam, the Northern Mariana Islands, Nauru, and Kiribati (Micronesian Islander Community, 2017). As of 2012, Marshallese is the third most commonly spoken language in the Salem-Keizer School District (Oregon Department of Education, 2014). Despite the population growth, MI participation in research is sparse, and there is paucity in MI represented fine-grained data in health-related literature.

A history of unethical research practices in the Pacific has led to MI community members’ reluctance and wariness to engage with researchers who are not familiar with the community’s culture (George, Duran, & Norris, 2014; Scharff et al., 2010). One of the largest and most devastating actions to the MI community occurred when the U.S. government tested nuclear bombs in a region of Micronesia that destroyed the way of life and ecological systems on the island and surrounding territories. From 1946 to 1958, the U.S. government conducted more than 60 nuclear weapons tests in the Marshall Islands, or the equivalent of over 7,200 Hiroshima-sized bombs (Letman, 2013). The largest nuclear weapons test occurred in March 1954, with over 1,000 times the strength of the bomb that destroyed Hiroshima (Letman, 2013).

The immediate and long-term impact of the bombings continue to haunt the MI community, in particular the Marshallese, today. The nuclear fallout resulted in coral rock, soil, and other debris becoming an inferno fireball and made intensively radioactive by the nuclear reaction (Simon, Bouvile, Land, & Beck, 2010). While the U.S. government relocated residents within the immediate bombing area (specifically the Bikini and Enewetak atolls of the Marshall Islands), the fallout from the testing reached farther locations the U.S. government did not anticipate (Simon et al., 2010). As a result, the Marshallese group within the MI community experienced a higher number of miscarriages and stillbirths and high rates of intellectual and developmental disabilities (Simon et al., 2010).

There is a limited amount of research on maternal and children’s health in general. In Hawaii, MI women from the Federated States of Micronesia and Republic of the Marshall Islands often experience late entry into prenatal care and do not engage in sexually transmitted disease screening, potentially leading to high infant mortality (Arakaki, Anderson, Yoda, & Samifua, 2004). In another study, researchers found that only 20% of MI women received prenatal care in the first trimester (Yamada & Pobutsky, 2009). This is similar to an urban area in the Pacific Northwest where Pacific Islander women including MI are less likely to access prenatal care in the first trimester than non-Latinx White women (62.7% vs. 24.3%, respectively; Multnomah County Health Department, 2015). In regard to the school system, Pacific Islander children have high absentee or drop-out rates due to family obligations (Ratliffe, n.d.). Pacific Islander children are more likely to experience poverty than non-Latinx White children (28.6% vs. 13.2%, respectively; Multnomah County Health Department, 2015). Researchers need to learn and understand community perspectives in order to properly address the health disparities within a minority community (Sullivan et al., 2001). Organizational MI leaders at the Micronesian Islander Community (MIC) organization expressed a need to explore the voices of MI as parent leaders.

The research team, consisting of a community-academic partnership, were aware of the circumstances and suspicions that the MI community would have. The academic partner was an academic nurse researcher (principal investigator) and two community research team members (of which one is another principal investigator, while the other is a MIC organization staff member). The community research team members were also certified community health workers (CHWs). According to the American Public Health Association (2019), “A community health worker is a frontline public health worker who is a trusted member of and/or has an unusually close understanding of the community served.” The purpose of our qualitative descriptive pilot study was to explore the perceptions and experiences of MI parent leaders with maternal and children’s health, the school system, and the influence of culture. This study was a part of a larger Health and Education Leadership program to build capacity with MI as parent leaders. MI parent leaders are community member residents within the local MI community. The Washington State University Office of Research Assurances determined that the pilot study (IRB # 17203-001) satisfies the criteria for Exempt Research at 45 CFR 46.101(b)(2).

## Methods

### Design

The MIC organization, a non-profit culturally diverse community-based organization in Oregon, and an academic nurse researcher from Washington State University College of Nursing, in the Pacific northwest region of the U.S. established a partnership in 2018. A community-based participatory research (CBPR) design was used to conduct this pilot study that involved collaboration of the above-mentioned research team members as partners. As a research team, we defined the study’s purpose; created the interview guide; recruited participants; facilitated group discussion; analyzed and interpreted data with the participants; and determined the next steps in our research partnership. The community and academic partnership is a long-term commitment that builds upon the foundation of trust, co-learning, and commitment (Israel, Eng, Schulz, & Parker 2012; Wallerstein, Duran, Oetzel, & Minkler, 2018).

### Participants and Setting

The MIC CHW staff community researcher (who identifies as Chuukese and speaks English and Chuukese) conducted outreach and recruited 11 MI parent leaders as participants who are known by the community research team members through their organizational network from an urban area of the Pacific Northwest region of the U.S. Of the 11 MI parent leaders, three decided not to participate due to a work schedule conflict. The inclusion criteria included: participants who self-identified as a MI parent with a child or had children less than eight years old including age zero and could speak and understand English or a MI language. All eight participants self-identified as Chuukese or Marshallese and all chose to communicate in English. One of the principal investigators (the academic nurse researcher who identifies as Vietnamese and speaks English and Vietnamese) obtained consent and provided the study information sheet. The participation size was sufficient for coding stability according to a methodological study by Hennink, Kaiser, and Marconi (2017). A meal and childcare was provided for the group discussion. Each participant received a $25 monetary gift card for remuneration.

### Data Collection and Analysis Procedures

#### Group level assessment includes illustrative storytelling

Micronesian Islanders are historically known to be an oral culture (Mitchell, 1970). Building capacity through sharing stories is a way to develop a long-lasting relationship within the community. The participants were MI parent leaders who participated in the data collection and analysis with the academic nurse researcher and the trained MI CHW staff community researcher using the steps in the group level assessment (GLA) method (authors described details about the steps later) that included illustrative storytelling (i.e., writing and drawing pictures while telling stories) and lasted 90 minutes in duration. Writing and drawing pictures can help with reflection on the intended meanings or interpretations including difficult things to talk about (van Wyk, 2011). In accordance with the GLA method, data collection and analysis occurred concurrently in real time with participants and two researchers as co-facilitators. The academic nurse researcher and the trained MI CHW staff community researcher co-facilitated a group discussion with seven MI parent leaders. Another parent leader participated individually in the data collection and analysis with the two research team members using the same GLA method steps. Due to a health reason, this MI parent leader was unable to participate with the other MI parent leaders. In offering the opportunity to participate as an individual, we removed a barrier from the parent leader’s participation in the study. We followed six steps in the GLA method: climate setting, generating data, appreciating perspective, reflecting, understanding the data, and selecting themes (^1^Nguyen-Truong, ^1^Fritz et al., 2018; Vaughn & Lohmueller, 2014). GLA illustrative storytelling provided timely and valid data.

In the *climate setting step*, co-facilitators sought to build trust with MI parent leaders while being culturally sensitive to create a comfortable atmosphere for sharing their stories. Co-facilitators asked participants to come up with group names to build community and collaborative efforts together. Participants came up with the final group names: Island Mix, Island Dream, D’ Dreamers, and Island Way. In the *generating data step*, the MI CHW staff community researcher provided a pack of assorted color pens and large adhesive flip chart papers. Then, the academic nurse researcher and MI CHW staff community researcher co-facilitated the discussions and used an open-ended, semi-structured interview guide with questions and prompt starters (see Box 1). The co-facilitators provided MI parent leaders with the interview guide as a handout. The co-facilitators stated examples on how they would refer to early learning and education as systems. Due to the exploratory nature of the pilot study, we started with broad questions and prompt starters to allow MI parent leaders to direct how they would like to share their stories through written words and drawings.

**Box 1** Open-Ended, Semi-Structured Interview Questions and Prompt Starters

Here are examples of early learning and education systems. We ask that you keep this in mind as you are sharing about your experiences and perceptions on the questions and prompts that follows after.

- Early learning: preschool, head start
- Education (formal schooling): elementary, kindergarten, first grade, second grade, third grade

What barriers do you perceive or have you, your family, or MIC experienced in early learning, education, or health care systems?

- The toughest barrier is …
- A concern is …

What desires do you as a Micronesian Islander parent leader have for early learning, education, or health care systems?

- In order for change to happen in early learning, education, or health care system, then what needs to be considered in regard to the Micronesian Island culture?
- The best thing that can be done as a change in early learning, education, or health care system is …

The co-facilitators asked additional follow-up and probing questions to encourage explanation and elaboration on responses related to the purpose of the study. For example, “Please tell us more about your experience when you said English language is a barrier,” “What are your thoughts on why MI women are not engaging in early prenatal care?,” and “Please tell us more about your experience regarding the interaction between a MI parent of young children and the school teacher.” Each MI parent leader transcribed their own data in this process through having shared their story by drawing pictures and writing the main points of their story directly onto the flip chart papers. Then each MI parent leader read their story aloud while pointing to the pictures. Although MI parent leaders had an opportunity to ask each other questions, they did not have questions. They verbalized agreement about having similar experiences and perceptions. The co-facilitators verified main points and drawings with MI parent leaders in real-time to assure trustworthiness of the data (Lincoln & Guba, 1985). The co-facilitators stated their understanding and interpretation of what the MI parent leaders said and MI parent leaders confirmed accuracy in understanding and interpretation. In the *appreciating perspective step*, the academic nurse researcher and MI CHW staff community researcher reviewed the main points and encouraged MI parent leaders to add anything to their stories that came to mind. In the *reflecting step*, participants quietly thought about what the responses and interpretations of the drawings meant as a whole and added any summative thoughts. In the *understanding the data step*, MI parent leaders identified common themes from the content with the two co-facilitators and arrived at consensus (i.e., written and drawn on the flip charts). In the *selecting themes step*, MI parent leaders selected and prioritized common themes with the two co-facilitators and the other principal investigator (the MIC Board Chair/CHW who speaks English) who joined in the selecting themes step and arrived at consensus. This allowed for verification through debriefing about the final themes to ensure that they align with the GLA illustrative storytelling method.

The academic nurse researcher and MI CHW staff community researcher who were co-facilitators independently wrote field notes of their observations. Then as a research team, the academic nurse researcher and the two community researchers/CHW discussed immediately following their impressions of the group process, interactions, and addressed questions. Reflexivity was used throughout the analytic process as a technique to address the influence of potential personal biases on findings (Rae & Green, 2016).

## Findings

### MI Parent Leader Characteristics

All eight MI parent leaders identified as female. All MI parent leaders reported coming from two islands to the U.S. and are Compact of Free Association (COFA) citizens. This included six MI parent leaders who were born in the Federated States of Micronesia, specifically Chuuk, and two who were born in the Republic of Marshall Islands. They spoke Chuukese and Marshallese as their native language, respectively. The COFA is an international agreement that established and governed the relationship between the U.S. government and the Micronesian nations [described earlier] in 1986 (McElfish, Hallgren, & Yamada 2015). The mean age is 35.13 years (range = 26–42 years), and the mean years lived in the U.S. is 12.63 years (range = 2–22 years). Most MI parent leaders (6) reported a college level education followed by high school (1) (one declined to answer). Nearly all (7) reported speaking English well followed by one who reported very well. Seven reported having employment. Most (5) reported being currently married and two reported being single. Most (5) reported having less than $15,000 as a total household income before taxes, followed by $15,000–30,000 (2) and $30,001–50,000 (1). MI parent leaders reported the following occupations: caregiver (3), interpreter (2), certified community health worker and program coordinator (1), and an auditor (1). All (8) MI parent leaders have health insurance. Most (6) reported having a state-sponsored health plan followed by private health insurance (2). Most (6) reported having a regular place of health care (primary care provider office, community health service center, or a county health clinic), whereas two seek health care at a free health clinic.

### Main Themes

We identified four main themes as follows: MI cultural identity, English language and MI culture disharmony, zero or delayed prenatal care, and uncertainty for the future of MI children with disabilities or developmentally delayed as children progress through the school system.

#### MI cultural identity

MI parent leaders described the importance of preserving the MI cultural way of doing things that reflect collectivism while living in the U.S. Participants talked about how their MI cultural upbringing is different from the U.S. culture. A MI parent leader said, “We do not think about ourselves. Everyone looks out for each other. This is our cultural upbringing. It is who I am.” Another said, “No matter where in the world I live, I will always live the island way. We came from the island... no matter what... we still carry our cultural way... The way my parents raised me. [I] took over what they taught me... the way to dress, the language... our different kinds of foods. Our cultural is pretty different. We still old fashion [traditional].”

Participants expressed longing to return to their island homes and missing their homelands. There seems to be a perception of the MI community being visitors instead of a resident in the U.S. As described by a MI parent leader, “A dream for our islands is what we learn in the U.S. hopefully will [be] able to give back to our community, islands.” Participants described living in two cultures simultaneously as being difficult because of the stressor of navigating between understanding U.S. customs that at times conflict with MI culture. MI parent leaders expressed the importance of building a sense of MI community to help community members who are struggling to adapt to the U.S. way of doing things. However, this is difficult in both visibility and connection efforts. “We come from different islands to the United States. Difficult to gather people but work together as parent leaders to gather our people together. We are all spread out [in this state].” They talked about being a shy culture who are not accustomed to speaking about issues. “We are shy. Do not ask questions.”

#### English language and MI culture disharmony

MI parent leaders described language as a substantial barrier in the MI community as parents are experiencing difficulty in their interactions with the children’s teachers and navigating the health care system. One MI parent leader said, “I’m respectful but limited understanding for English language might put me in a situation where others might think I’m not respecting.” This parent leader drew a portrait of herself along with the statement, “I am respectful”, and expressed having courage to speak up about this struggle. Participants described how limited English has made them appear to not care or not following directions. They expressed that terms are confusing for MI community members. One MI parent leader said,

[My parents] were just silent and did not really understand what was going on. Difficulties understanding each other [teacher and parents]. Silent – they [parents] didn’t really understand. More of nodding and shaking their head. I am sure they wanted to say more but they couldn’t say whatever they wanted to say and didn’t know the words what to say.

That MI parent leader described that she could not join a sport or take school pictures due to her parents not understanding English and said how they did not read letters sent from school such as permission forms. MI parent leaders expressed the need for the school to outreach to families using a culturally and language sensitive approach to education to parents regarding opportunities for children to engage in school related activities.

MI parent leaders talked about how there are not enough culturally-specific language interpreters for the MI community. They expressed that it would be helpful for hospitals and clinics to have their own interpreters from different islands because of the diversity within the larger Pacific Islander group. For example, the MI community is a subgroup within subgroups with different languages. MI parent leaders talked about language as a barrier to immunization and how vaccinating children is expected for entry into the school system unless there is documentation for exemption: medical, religious, or not wanting to vaccinate (personal exemption) according to Oregon State (Oregon Health Authority, n.d.). MI parent leaders also recommended offering different island language courses within public schools and to outreach and provide free English classes for MI.

#### Zero or delayed prenatal care

MI parent leaders described prenatal care as being an unfamiliar early care process within the MI community. They talked about how MI women who are pregnant often do not prioritize having regular check-ups by a health care provider and when they do, it is in later months of the pregnancy. One parent leader said, “In the island, wait until seven to eight months later.” Another MI parent leader drew a picture representing a community member who is pregnant asking, “What is prenatal?”, and talked about how prenatal care is really not being done by MI community members. One described, “…when it comes to a girl becoming pregnant, they [MI community] think checking on them daily at the start [is] not supposed to prioritize. Do not look at prenatal…Back at home [island], they…do not check on daily or weekly. If the babies are going to make it, they gonna make it. They use God, their religion. If God said that the baby is to be healthy, then they just wait.” A MI parent leader described that this belief within the MI culture regarding religious faith appears to be passed down from generation to generation from the island. MI parent leaders expressed the need to talk and outreach about the importance of prenatal care. This also included their concern for “young motherhood” who are adolescents who do not seem to be receiving support because of family disapproval of becoming pregnant at a young age. One MI parent leader described having urged MIs to go see their health care provider for regular check-ups due to her personal experience of giving birth to a stillborn baby.

#### Uncertainty for the future of MI children with disabilities or developmentally delayed as they progress through the school system

MI parent leaders expressed challenges in understanding what kind of programs are available in early learning and elementary school that would fit the basic needs for children with disabilities or developmentally delayed from that of the general population of children. Some MI parent leaders discussed their frustration with respect to the future of MI children with disabilities when they are adults, and the need to address this earlier on while they are young. This can be further difficult for families with children with disabilities who are not U.S. citizens as the availability of state and local resources is based on citizenship in the U.S. As described by a MI parent leader,

As a Community Health Worker, I’ve been reaching out with families that needed help within our Micronesian [Islander] community [in Oregon]. There are families that have a kid who is disabled. [For example], I helped them find a school that is suitable for the kid. The frustration is [when] the kid who is disabled have a different status. He is a non-U.S. citizen. When you are a non-U.S. citizen in the U. S., it’s hard to find resources to help with the disabled. What I found out is that it depends on [the] state. For instance, in Hawaii, Washington, and California, they have funding that help the non-U.S. citizen within their State. In Oregon, there is none.

MI parent leaders expressed that supporting children’s development earlier on in the school system needs to be a collaborative effort with the teachers. This can be challenging when MI parents feel that that they are not being included or not having their voices heard by the educators in the school system combined with not understanding how to navigate the school system regarding what it means to have a child that is *school ready*. Being uncertain of the pathway regarding what school readiness entails for promotion from early learning to kindergarten to subsequent grade levels was a concern including what the roles and responsibilities are for teachers and parents regarding preparedness. A MI parent leader described an example of her being asked by a MI community member to help advocate.

A single parent with a 5yr old son… don’t know how to talk, still in diapers, and cannot comprehend what he learned… he was… developmentally delayed [and enrolled] in the developmental kindergarten. The school staff including the principal… moved the 5yr old kid to the regular kindergarten… Mom was very upset. During a meeting [scheduled by the mom], … the kindergarten teacher told mom… not ready to promote to first grade due to the delay. I asked… what they base their promotion on when they [had] moved the child [previously] … they use the other teacher assessment on the child during classroom hour… the child meet the cut-off point in the system so he was not eligible to any special education. This is very sad to the mom… knows her child better… The school decided to hold the child back in kindergarten one more year.

MI parent leaders talked about needing to engage with MI community members in a personal way to connect the message. As described by a participant who was approached by a teacher,

I had a son who was in elementary school and saw a paper about preschool [posted on a wall at the elementary school]. I have a younger son but walked by the paper. My sons’ elementary teacher talked with me asked me how old is your son [referring to the younger son] and spoke about the preschool. After, I went ahead and enrolled my son approaching four [years old] into preschool.

That MI parent leader said that the elementary teacher knew her older son, took the time to reach out to her regarding her younger son, and how this experience helped to connect with her son’s teacher while learning about the importance of preschool.

## Discussion

MI parent leaders shared their hopes and feelings centered on their struggles with U.S. culture, learning English, their understanding of early access to health care for prenatal care, and the importance of receiving early and quality childhood education.

In the current pilot study, MI parent leaders discussed being a shy culture who are not accustomed to speaking about issues and not thinking about one’s self, but rather looking out for others. Caring for others is a common issue in many immigrant communities. Cultures with a strong collectivist perspective, such as MI culture, are concerned more with fulfilling family obligations (Hardway & Fuligni, 2006). There is less individualism as seen in more Westernized cultures. As such, supporting family is more important. Micronesian Islanders maintain relationships with one another through participating in community gatherings. This includes attending family and community events, including funerals, birthdays, and weddings, and often entails providing food, money, and manual labor such as decorating the location (Ratliffe, 2010). These relationships are important for maintaining community connections. Some activities are not optional and cost money. These are usually funerals or other large community activities mandated by other MI community members. Our findings suggest that often when a family member needs something, someone else usually within the family provides the needs.

Findings point to MI parent leaders’ concern regarding how language barriers made them appear as though they did not care or follow directions by health care providers and children’s teachers. This is similar to other studies that evaluated MI access to health services. Pobutsky, Krupitsky, and Yamada (2009) found that many MI reported problems accessing the health and social service systems, primarily due to language barriers. Often, MI who do not speak English rely on a family member to serve as their interpreters. Finding quality and trained interpreters is difficult to obtain (Hattori-Uchima, 2017). The inability to communicate with health care providers could lead to mistrust due to misunderstandings. The MI, relying on a family member or improperly trained interpreter, may receive incorrect information and in the confusion of receiving incorrect medical information, becomes frustrated with the health care provider. Similarly, educators are supposed to understand how a child’s culture influence their learning. Ratliffe (2010) reported that teachers sometimes judge families negatively due to not understanding the culture (DeCastro-Ambrosetti, 2005). For educators to properly engage with MI parents, educators need to understand or be willing to learn about the MI community.

Cultural beliefs appeared to be passed down through generations from the MI island homeland influencing whether to engage in prenatal care. There are limited health care resources in Micronesia. Yet, MI are aware of local medicine that community rely on while living on the islands. Due to the limited number of resources, MI often do not seek prenatal care. MI pregnant persons then come to U.S. without seeking additional care. Often, their first time visiting the health care provider for a prenatal care visit is when they are about to give birth. Our findings differ from a previous study. Hattori-Uchima (2017) study with Chuukese migrant women in Guam reported that although women did not engage in preventative care services, they did engage in prenatal care when they were pregnant. Ayers et al. (2019) found that Marshallese women expressed the desire to incorporate culturally specific prenatal care support. Similarly, MI parent leaders shared their stories of utilizing local medicines during pregnancy on the islands. Health care providers should play a critical role by asking pregnant MI parents if there are specific cultural practices that the expectant parent would like to incorporate during their pregnancy. Furthermore, community outreach and education are important to ensure that expectant MI parents understand the value of prenatal care. For example, a MI parent leader often did outreach with local women early in their pregnancy. Our findings point to the importance of providing wide-spread culturally specific outreach and interventions focused on increasing access to increase utilization of prenatal care services.

Micronesian Islander parent leaders expressed uncertainty regarding what preparedness entails by teachers and parents for promotion from early learning to subsequent grade levels. There is scant information on family obligations in MI culture and whether MI parents realize the importance of their role on their children’s education in U.S. schools. Family obligations may impact a child’s education when MI parents or caregivers miss meetings. Similar to our findings, another researcher found MI parents want their children to succeed (Heine, 2002). However, our findings suggest that MI are often not aware of the programs and resources available to them. MI come from islands and live on subsistence lifestyles through growing their own food and fishing. However, in the U.S., it is impossible to grow their own food, and MI parents may need to work several low wage jobs to earn enough income (García Coll et al., 2002). Previous researchers found that MI families may live with others in crowded conditions to make rent (García Coll et al., 2002). These added stressors can impact family dynamics and make it more difficult for MI parents to participate in school-related activities for their children (García Coll et al., 2002).

Educators and local community organizations need to work together to meet with MI communities to discuss the importance of early learning programs and the importance of parent engagement. This could include culturally specific consideration in the discussion about attending parent-teacher meetings or being available to meet with parents outside of regular scheduled hours because MI families may be occupied with a multitude of obligations. Educators could work with MI parents to ensure a mutual understanding on roles and responsibilities regarding school readiness for optimal educational attainment. This requires an understanding and appreciation of the culture and recognizing that the children may be second generation immigrants.

We learned from the pilot study that MI parent leaders identified prenatal care access and utilization and early education as important issues for the community to learn and understand the importance. This included MI parent leaders acknowledged that they learned the importance of obtaining access to prenatal care and utilizing health care services during their pregnancy through their own experiences with the health care system. They mentioned ensuring receiving support that is culturally specific and appropriate to their community within the MI community, such as providing quality trained interpreters who spoke their language(s) and health care providers who knew about their culture and community. The MI parent leaders also discussed the importance of early childhood education, for example early *head start*, and learning how to navigate the educational system with children who are developmentally delayed or who have learning disabilities. The findings suggest that future research is needed in examining differences in groups within the MI community in relation to culturally specific prenatal care and whether the educational attainment level of a MI parent impacts their ability to engage in a meaningful way with their children’s education experience. Building leadership skills among MI parents may be the first step towards increasing recognition by health care providers and educators about the MI community. As MI parent leaders continue with building their leadership skills, it is possible that they will take initiative to build momentum towards building stronger relationships with healthcare providers and educators and learning to serve as advocates for their community while educating others about the MI way of life. In doing so, MI parent leaders can organize the MI community, health care providers, and educators through building new relationships to educate and co-learn with others about the MI reality while also serving as advocates for the MI community.

There are some limitations. This qualitative descriptive pilot study was conducted with MI parent leaders in an urban area in Oregon within the Pacific Northwest of the U.S. MI parent leaders provided information from their experiences and perspectives and within the MI community including from parents from Chuuk and the Marshall Islands. There may be different findings for other MI groups. Illustrative storytelling as an approach may not fit with everyone. Future research is needed to explore perceptions and experiences of MI with school and health systems in urban and rural settings as the experiences may vary.

## Conclusion

MI parent leaders shared their perceptions and experiences regarding the school system and prenatal health care and the influence of MI culture. Findings point to how MI parents struggled with understanding how to enroll into school readiness programs to prepare their children for the U.S. public education system and the importance to early prenatal health care. During the storytelling, MI parent leaders demonstrated awareness and strength of their voices, and the need to learn and know the resources that are available to families. At the same time, there are concerns and health care providers including nurses and school officials need to play an important role and should consider the concerns that hamper proper care and guidance in the importance of early education and prenatal care. This includes having a culturally specific understanding of the MI community, their needs, culture, and language barriers. This article can serve as a potential guide for others who want to work with MI communities.

## Acknowledgments

The authors are appreciative of the anonymous peer reviewers for assistance.

## Declaration of Conflicting Interests

The authors declared no potential conflicts of interest with respect to the research, authorship, and/or publication of this article.

## Funding

Dr. Jacqueline Leung, JD, MS, CHW, Dr. Connie Kim Yen Nguyen-Truong, PhD, RN (Alumnus PCCN), and Kapiolani Micky, BA, CHW, received the Health and Education Fund Impact Partnerships #18-02376: Northwest Health Foundation, Meyer Memorial Trust, Kaiser Permanente Northwest, Care Oregon, and Oregon Community Foundation.
